# Enhancing Cognitive Functions of Older Adults With Software Robot: Longitudinal Exploratory Field Study

**DOI:** 10.2196/75308

**Published:** 2026-06-24

**Authors:** Byunghun Yun, Bohee Kim, Hyunjeong Ko, Jinsung Kim, Bori R Kim, Whani Kim, Soyoon Park, Jinwoo Kim, Jee Hang Lee, Geon Ha Kim

**Affiliations:** 1 HCI Lab, Department of Cognitive Science, Yonsei University, Seoul, Republic of Korea; 2 Digital Therapeutics Research & Development, HAII Inc, Seoul, Republic of Korea; 3 Cloud Service Team, LG Electronics, Seoul, Republic of Korea; 4 Department of Human-Centered AI, Sangmyung University, Seoul, Republic of Korea; 5 Ewha Medical Research Institute, Ewha Womans University College of Medicine, Seoul, Republic of Korea; 6 Department of Neurology, Ewha Womans University College of Medicine, Ewha Womans University Mokdong Hospital 1071, Anyangcheon-ro, Yangcheon-gu, Seoul, 07650, Republic of Korea, 1 82-2-650-5298; 7 Institute for Advanced Intelligence Study, Daejeon, Republic of Korea

**Keywords:** mobile phone, mild cognitive impairment, chatbot, aging, dementia

## Abstract

**Background:**

The global prevalence of dementia continues to rise and demands scalable, nonpharmacological interventions. Digital cognitive training has expanded, but many older adults, who have limited digital literacy, struggle to sustain use. We designed an intervention that integrates social interaction, reward-based engagement, and an artificial intelligence (AI) conversational agent, which aims to reduce these barriers and support continuous participation.

**Objective:**

This study examined whether a 12-week digital cognitive training program improves cognitive function in older adults. It also tested whether a group chat service, which enables interaction among participants and with an AI agent, increases engagement and social support.

**Methods:**

We recruited 133 participants (mean age 64.75, SD 6.76; range 55-75 years) who had no diagnosis of dementia. All participants used the *Care & Cure* program for 12 weeks. The program includes an AI chatbot (*Sammy Talk*) and a group chat service (*Our Town*), which supports peer interaction. We measured cognitive function using the Korean Mini-Mental State Examination—Version 2 (K-MMSE-2). We also assessed degrees of social support (Medical Outcomes Study—Social Support Survey), depression (Short Form Geriatric Depression Scale—Korean Version), and engagement (Twente Engagement with eHealth Technologies Scale), and analyzed usage logs to examine participation patterns.

**Results:**

Participants showed improved cognitive function after the intervention (Hedges *g*=0.350, *P*<.001). Active users (n=67), who engaged more frequently with the program, showed greater improvement than nonactive users (n=66), especially among those who had lower baseline cognitive scores (Hedges *g*=0.523, *P*<.001). Social support increased, particularly emotional and informational support (*t*_132_=−6.509, *P*<.001). Participants reported higher engagement (*t*_132_=2.008, *P*<.05) and lower depression scores (*t*_132_=3.093, *P*<.01). Regression results showed that group chat participation, which promotes interaction with the AI agent, increased engagement in cognitive training (*t*_131_=12.395, *P*<.001). Increased engagement was associated with higher social support (*t*_131_=4.102, *P*<.001) and improved cognitive function (*t*_131_=2.467, *P*<.05). Cognitive training alone did not produce a significant effect. Participants showed low adherence, which indicates a need for strategies that sustain long-term use.

**Conclusions:**

The *Care & Cure* program improved cognitive function and strengthened social support in older adults. Social interaction, which increases engagement, played a central role. These findings suggest that digital cognitive interventions should incorporate social mechanisms to achieve meaningful effects.

## Introduction

### Background

The global prevalence of dementia is rising, with recent estimates indicating that approximately 50 million people currently live with dementia. It is estimated that the number will increase to 152 million by 2050 [1]. Dementia has a significant impact on personal, family, and economic levels, costing the world about 1.3 trillion dollars [2]. Prevention is essential for dementia management. Among nonpharmaceutical interventions, digital cognitive training has become more popular because prevention is essential for dementia management. Among nonpharmaceutical interventions, digital cognitive training has gained increasing popularity as long-term clinical studies demonstrate that older adults who engage in regular digital cognitive training could delay dementia onset by preserving memory, attention, calculation, language, visuospatial, and executive functions [3-7]. This preservation helps build cognitive reserve that protects against age-related cognitive decline [8,9]. In practice, traditional cognitive training programs are constrained by several limitations. These include (1) limited accessibility for older adults who have mobility issues, (2) heavy reliance on social workers, and (3) substantial financial costs, which are incurred by individuals through personal expenses for participation and by society through health care and social services for people living with dementia [6,10,11].

Many studies suggest that digital cognitive training programs with remote access can overcome these barriers [3-7]. These programs demonstrate greater scalability since they can reach a larger number of users simultaneously without requiring additional resources or staff. For example, while traditional cognitive training requires more therapists or social workers for each new group of users, digital cognitive training programs can be used by many more users with minimal additional cost. This efficiency in scaling makes them more cost-effective, creating a more sustainable and widely accessible option [12-14].

However, current digital training programs have limited accessibility from home mainly because of inadequate consideration of older adult users’ digital literacy [15,16]. Low digital proficiency among older adults restricts their access to digital training programs [17]. Even older users with the ability to use digital training programs at home and with the necessary technological infrastructure often struggle with prolonged engagement [18]. Older adults face many challenges, including physical limitations, difficulty processing information, and digital service interfaces that are not easy to use [19]. These barriers worsen clinical results as the disease progresses, increase medical costs, and impose a greater patient burden [20].

Several solutions address these challenges, including simplified user interfaces that reduce cognitive load, gamification elements designed to promote user engagement, personalized digital literacy support, sending push notifications, using chatbots, and using tools to help people connect with others. These features (1) enhance usability, (2) reduce cognitive demand and screen fatigue, and (3) ultimately improve compliance and clinical outcomes [21]. Among these solutions, chatbots and group-based interventions have shown relatively strong potential for enhancing user engagement and effectiveness in digital cognitive training programs demonstrated by numerous studies [21-23].

Conversational agents, also known as chatbots, have the potential to resolve these barriers [24]. A substantial number of studies have shown that conversational agents used for health care interventions elicit moderate to high levels of participant satisfaction and acceptance, largely due to social support features [25-29]. These agents show promise to advance patient-centered care by supporting users in managing their own health [24,25]. Additionally, group-based interventions enhance program engagement by fostering interaction among members [27]. It is widely accepted that conversational agents provide effective social support when integrated with group interventions [28,29]. Two recent studies have validated the effectiveness of a fully automated mobile conversational agent in providing social support [28,29]. In health care settings, the Shared Medical Appointments model, which integrates group-based interventions, continues to gain wider acceptance as a way to improve patient engagement in treatment programs [30-33].

Under this circumstance, we propose *Care & Cure*, a chatbot-based service. The *Care & Cure* service offers 15 different cognitive training programs through *Sammy Talk*, along with a group chat feature called *Our Town*, where participants can interact with one another. This group interaction is intended to improve user engagement with *Sammy Talk*. At the core of this service is *Sammy*, a software robot that delivers personalized cognitive training and fosters social support through conversational interactions. By integrating these features, *Care & Cure* aims to meet both cognitive training and social needs of older adults.

### Objectives

The primary objective of this study is to evaluate whether the *Care & Cure* program enhances cognitive function in older adults. The secondary objective is 2-fold: (1) to assess how social support impacts cognitive function and (2) to determine whether increased engagement with *Our Town* encourages greater usage of *Sammy Talk* and interaction with the artificial intelligence (AI) agent. To achieve these objectives, we analyzed the utilization patterns of both *Our Town* and *Sammy Talk* to assess their effects on cognitive function and social support. As an additional evaluation beyond the primary focus on cognitive functions, we examined the effect of social support functionality embedded in both *Sammy Talk* and *Our Town* on emotional well-being, specifically depressive symptoms, which emerged as a complementary benefit to the cognitive improvements.

## Methods

### Study Design

This was a single-arm, prospective, exploratory study. The protocol and all related materials were approved by the Yonsei University Institutional Review Board (IRB; 7001988‐202401-HR-1397-06) prior to the study’s initiation.

### Study Recruitment

We recruited participants from Suncheon, a midsized city in South Jeolla Province, South Korea, with a population of approximately 280,000. The city’s older adult population reflects South Korea’s aging demographics, with about 20% of residents aged 65 years or older [34]. Suncheon is recognized for facilities that support senior citizens through welfare centers, nursing homes, and silver towns that provide housing and health care services for older adults [35] (see Section 1 in Multimedia Appendix 1 for more details).

*Suncheon Nonghyup*, a key institution in the city, provides substantial support to the older adult community. Its well-established network facilitated the recruitment process (see Multimedia Appendix 1, Section II for more details). The central office of the farmers’ cooperatives distributed formal recruitment documents to local branches. Through this network, we recruited participants who met the inclusion criteria: adults aged 55 and 75 years and proficient in using a smartphone. All participants provided written informed consent prior to participating in the study.

### Inclusion and Exclusion Criteria

The inclusion criteria were adults (1) aged 55-75 years who could read and write Korean (for more rationale, see Multimedia Appendix 1, Section III) and (2) owned a smartphone with text typing proficiency. We excluded adults who (1) lacked formal education or were illiterate, (2) had severe visual or auditory impairments, and (3) experienced difficulty using a smartphone.

Prior to recruitment, we evaluated these criteria through a preliminary screening survey. Participants provided demographic information, including age, educational background, and literacy levels. They also demonstrated smartphone proficiency by performing basic tasks such as navigating the interface and sending messages. Additionally, the survey assessed visual and auditory impairments. This screening process identified participants who met all study requirements.

### Intervention

Our intervention delivered cognitive enhancement games specifically designed by neurologists through 2 key components on the *KakaoTalk* platform: *Sammy Talk*, a conversational agent providing cognitive games, and *Our Town*, a group chat feature. We chose *KakaoTalk* because more than 70% of South Korea’s senior population use it [36]. The *Care & Cure* program, designed to prevent dementia, integrates both components into this widely used messaging platform.

**Figure 1.**
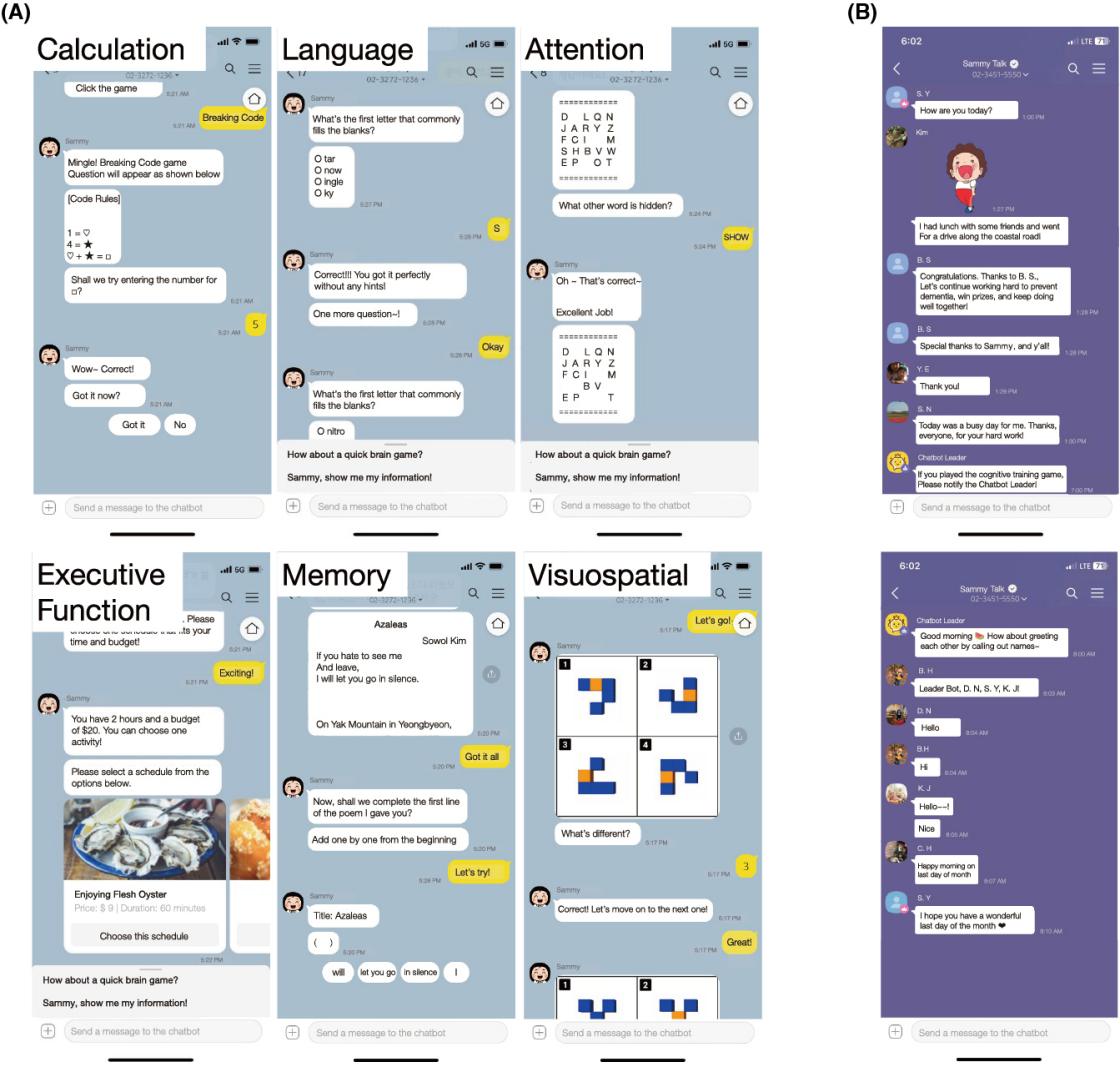
Overview on the proposed application. (A) Snapshots of *Sammy Talk* represent different cognitive domains in the following order: Calculation, language, attention, executive function, memory, and visuospatial function. (B) Snapshots of *Our Town*.

- A software robot, *Sammy*, functions as a conversational agent providing cognitive training through chatbot interactions. Conversational agents, which have demonstrated effectiveness in remote treatment delivery [37], support participants with communication difficulties through verbal or textual interactions [38,39] and are widely used for purposes such as memory enhancement [40] and CBT-based interactive tools for alleviating depression and anxiety [28]. For this reason, we developed *Sammy* as a conversational agent to offer 15 training games targeting 6 cognitive domains: calculation, language, attention, executive function, memory, and visuospatial processing [41-52] (Figure 1A; see Multimedia Appendix 1, Section IV). The game difficulty adapts across 5 levels as participants provide correct answers in *Sammy Talk*. Participants receive 4 games for cognitive improvement at 8 AM. A detailed description is available in Multimedia Appendix 1, Section IV.
- *Our Town* provides a virtual space where participants interact through group chat, promoting regular engagement with other members. Each group includes more than 5 members, and they receive notifications, and the members are expected to interact 3 times a day. An AI chatbot integrated into *Our Town* facilitates these interactions by moderating the group, encouraging participants to engage in *Sammy Talk*, and inquiring about their daily gaming activities and general well-being. Participants receive feedback and rewards based on their daily, weekly, or monthly game completion rates (Figure 1B).

The *Sammy Talk* chatbot functions as an autonomous digital facilitator, delivering morning greetings and encouraging cognitive training activities. The chatbot sends automated prompts 3 times a day (8 AM, 1 PM, and 7 PM) in *Our Town* to promote user engagement with digital cognitive games and ensure task completion. To complement *Sammy Talk*’s limited autonomous functions, a human facilitator motivates less active members, monitors individual participation, and provides personalized feedback with external rewards. The facilitator tracks participant messages in *Our Town* and monitors game performance through an administrative website.

For rewards, the group with the highest weekly participation rate receives gift cards worth KRW 5000 (approximately US $3.70, based on the exchange rate at the time of the study) for each participant and a group certificate. Additionally, when all group members complete their required 4 daily games, they receive restaurant gift cards worth KRW 10,000 per participant (approximately US $7.40, based on the exchange rate at the time of the study) for offline meetups. This integrated approach, combining an autonomous chatbot and human facilitation, fosters an environment that actively encourages engagement and consistent participation in cognitive training.

The interactions in *Our Town* exhibit straightforward structures with moderate turn-taking patterns. A daily interaction usually begins when the chatbot sends an automated morning greeting, which prompts individual responses from group members. These responses emerge sequentially, as members choose either personalized greetings or emoji-based communication. The exchanges primarily focus on 3 elements: mutual well-being inquiries, weather-related small talk, and positive message sharing. The structural simplicity of these interactions, which consist of brief greetings and basic phrases, ensures comfortable participation. Members demonstrate varied communication preferences: some address each participant individually, while others prefer concise responses or emojis. Each interaction typically consists of 5-10 turns, which reflects varying engagement levels. The uncomplicated, conversational nature of *Our Town* creates an accessible environment that accommodates diverse schedules and active communication styles.

A 2-day educational session took place at the *Suncheon Nonghyup* headquarters to establish baseline training. During this session, participants received an introduction to both the *Sammy Talk* program and *Our Town* group chat, alongside a comprehensive guidebook. The research team provided telephone instructions for those unable to attend in person. Subsequently, all participants received a text message with the program access link. The team installed *Sammy Talk* and *Our Town* directly onto the smartphones of session attendees.

### Outcome Assessment

The evaluation included 4 areas: cognitive function, emotional well-being, participant engagement, and application usage. We used 2 cognitive assessment scales: the Korean-Mini Mental State Examination—Version 2 (K-MMSE-2) [53] (see Multimedia Appendix 1, Section V for more details). These assessments took place at baseline and within 7 days after the 12-week intervention. The K-MMSE-2 uses a 30-point scale, where scores of 24 or above indicate normal cognition, 18‐23 suggest mild cognitive impairment, and below 17 indicate severe cognitive impairment. For emotional function assessment as a supplementary outcome, we used the Short Form Geriatric Depression Scale—Korean version (SGDS-K) [54] and the Medical Outcomes Study–Social Support Survey (MOS-SSS) [55] (see Multimedia Appendix 1, Section V for more details). The SGDS-K uses a 15-point scale, where scores of 5 or below indicate normal mood, scores of 6‐9 suggest moderate depression, and scores of 10 or above indicate severe depression. The MOS-SSS ranges from 19 to 95 points, with higher scores indicating stronger perceived social support. Changes in depression symptoms and social support served as secondary outcomes, with assessments occurring pre- and postintervention.

In addition to these, we measured participation willingness using the Twente Engagement with eHealth Technologies Scale (TWEETS) [55] (see Multimedia Appendix 1, Section V for more details), which uses a 9-point scale where higher scores indicate greater engagement willingness. The analysis on app engagement relied on log data acquired from the administrative websites of both *Sammy Talk* and *Our Town* (see Multimedia Appendix 1, Section V for more details). For *Sammy Talk*, engagement assessment included play frequency and duration across daily, weekly, and monthly intervals. For *Our Town*, engagement metrics comprised the number of chat exchanges and user-agent interaction duration across identical time intervals. The overall usage metrics included total *Sammy Talk* hours and *Our Town* chat bubble counts throughout the intervention period (Table 1).

**Table 1.** Measurement tool.

Category	Measurement		Standard
Cognition	Cognitive function	K-MMSE-2^a^	30
Emotion	Depression	SGDS-K^b^	15
Emotion	Social support	MOS-SSS^c^	19
Willingness to participation	Participation	TWEETS^d^	9
Log data	User engagement	Amount of time of cognitive enhancement game	Minute
Log data	User engagement	Number of interaction in group chat	Number

a K-MMSE-2: Korean-Mini Mental State Examination—Version 2.

b SGDS-K: Short Form Geriatric Depression Scale—Korean version.

c MOS-SSS: Medical Outcomes Study–Social Support Survey.

d TWEETS: Twente Engagement with eHealth Technologies Scale.

We arranged in-person meetings for pre- and postsurveys based on practical and methodological considerations: participant age preferences, assessment administration requirements, facility availability, and geographical accessibility. The target population, aged 55‐75 years, typically preferred face-to-face surveys over online formats. The *Suncheon Nonghyup* branch offered appropriate facilities, including halls and meeting rooms, for administering the K-MMSE-2, which required direct facilitator-participant interaction. The branch’s proximity to participants’ residences enhanced accessibility. Facilitators conducted presurveys during *Care & Cure* program introduction sessions, while postsurveys coincided with reward collection visits, ensuring effective data gathering.

### Study Procedures

**Figure 2.**
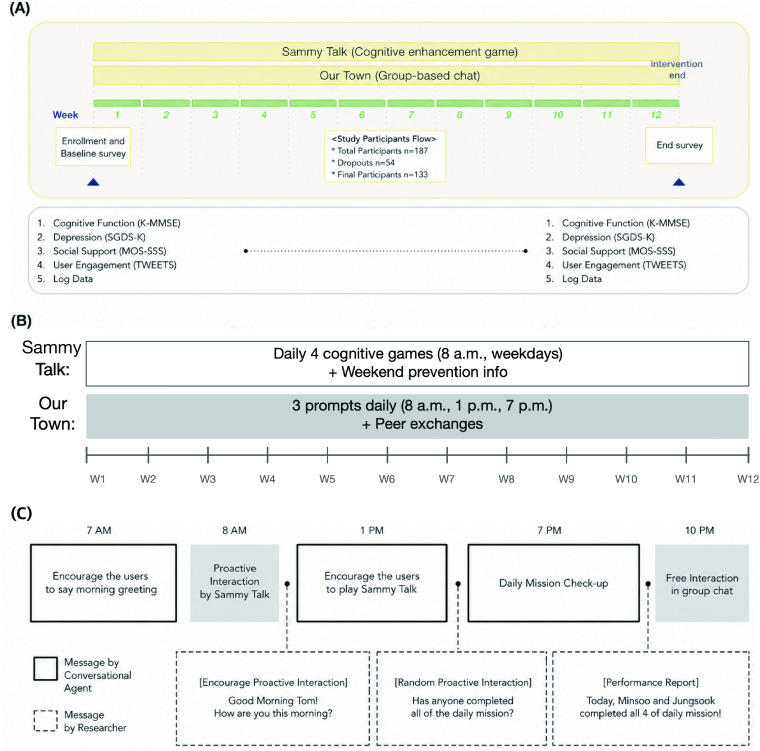
Overview of the study procedures. (A) Measurement tool according to the experimental procedure. (B) Timeline of participant engagement across *Sammy Talk* and *Our Town* during the 12-week intervention (C) Facilitator interactions in *Our Town*. CIST: Cognitive Impairment Screening Test; K-MMSE-2: Korean-Mini Mental State Examination—Version 2; MOS-SSS: Medical Outcomes Study–Social Support Survey; SGDS-K: Short Form Geriatric Depression Scale—Korean version; TWEETS: Twente Engagement with eHealth Technologies Scale.

We conducted the human subjects experiment for 12 weeks (Figure 2A). During this period, 133 participants (1) engaged in daily message exchanges through *Our Town* and (2) completed 4 daily sessions of *Sammy Talk*. Each day at 8 AM, participants received their daily activity instructions. Weekday activities consisted of 4 preselected games, whereas during weekends and holidays, the system delivered dementia prevention information and notifications at 8 AM. We conducted the 12-week intervention in a structured sequence of activities. The flowchart illustrates how customized cognitive training in *Sammy Talk* and group-based interactions in *Our Town* progressed in parallel and reinforced each other (Figure 2B). Participants maintained *Sammy Talk* engagement throughout the 12-week period. All participants completed these activities in their homes (Figure 2C).

### Statistical Analysis

We analyzed data from 133 participants using SPSS (version 28.0; IBM SPSS Inc). All participants completed the 12-week intervention, which included cognitive training games on *Sammy Talk* and the group chat platform *Our Town*. The study divided participants into 2 groups based on their engagement levels: active users (n=66) and nonactive users (n=67). Total usage included both *Sammy Talk* (which counted the number of games played) and *Our Town* (which counted the number of group chat posts). The median value of 65 sessions served as the dividing point: participants who recorded more than 65 sessions were classified as the active group, and those who recorded 65 sessions or fewer were classified as the nonactive group. We applied a “50% of the time” threshold, which ensured an even distribution between the 2 groups. This approach aligns with the methodologies used by Horwitz et al [56], who categorized participants into top and bottom 50% engagement groups in digital mental health interventions. This approach was also supported by Yardley et al [57] and Bucci et al [58], who identified that higher engagement correlated with improved clinical outcomes.

For comprehensive understanding of the impact of the training regarding the K-MMSE-2 scores, we additionally conducted a sensitivity analysis that categorized participants into lower (n=66) and higher (n=67) K-MMSE-2 groups, using baseline K-MMSE-2 median and SD. Participants with baseline K-MMSE-2 scores ≤27, which represented the median score, were categorized as the lower K-MMSE-2 group, while those who scored >27 were categorized as the higher K-MMSE-2 group. We also applied an alternative classification: participants whose baseline scores fell more than 1 SD below the mean were classified as having lower K-MMSE-2 status.

The analysis consisted of several components. Descriptive statistical analyses provided demographic information. Paired-sample *t* tests identified significant differences in cognitive function, emotional status, and participation between pre- and postintervention periods. Additional paired *t* tests compared outcomes between lower- and higher K-MMSE-2 groups and between high- and low engagement groups. Path analysis, through simple regression analyses, examined how group interventions affected *Sammy Talk* engagement, social support improvement, and cognitive function. Repeated-measures ANOVA assessed the mean and variability of daily *Sammy Talk* and *Our Town* usage across 3 months. Descriptive statistics supplemented this assessment for detailed data interpretation.

### Ethical Considerations

This study was approved by the Yonsei University IRB (IRB no. 7001988‐202401-HR-1397-06). All participants provided written informed consent prior to participation. The collected data were fully anonymized and securely stored, with access restricted to the research team. Participants received small incentives, such as gift cards worth KRW 5000 (approximately US $3.70) and restaurant vouchers worth KRW 10,000 (approximately US $7.40), based on the exchange rate at the time of the study, as compensation for their participation.

## Results

### Baseline Characteristics of the Participants

**Figure 3.**
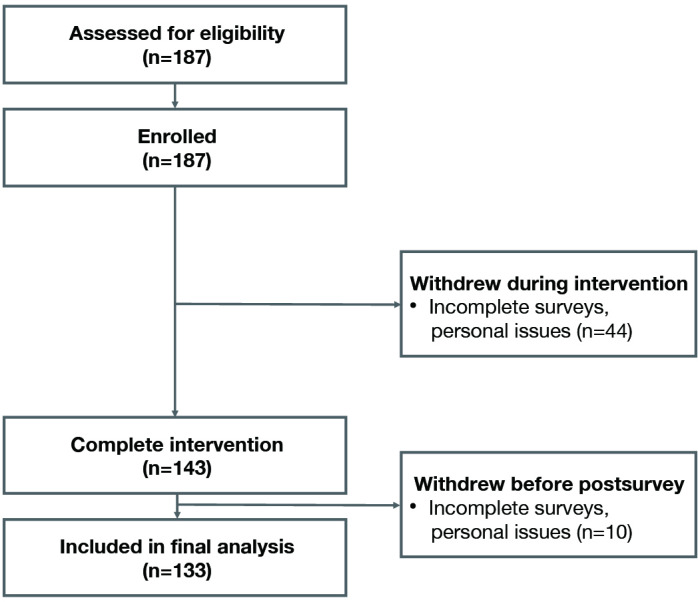
Flow diagram of study participants.

Initially, we recruited 187 participants for the study. Of these, 44 participants withdrew during the intervention period from the primary measure because they did not complete the survey or due to personal circumstances, leaving 143 participants. After the 12-week intervention, 10 more participants withdrew for similar reasons before completing the second measure, resulting in 133 participants available for final statistical analysis. Participants who did not complete the postintervention survey (n=54) were excluded from the final analysis. No statistical adjustments were applied for missing data from participants who withdrew before completion. Therefore, all statistical analyses were conducted using complete cases only. Figure 3 illustrates the flow of participant recruitment, retention, and exclusion throughout the study.

These 133 participants ranged in age from 55 to 75 years (mean 64.76, SD 6.76 years). Based on previous studies reporting medium effect sizes (Hedges *g*=0.5) and using G*Power (α=.05, power=0.80), we estimated that a minimum sample size of 128 would be required. This estimate is also consistent with the numbers of participants actually analyzed in similar studies involving older adults (100, 103, 145, 132, and 180 participants, respectively) [59-63]. To account for a potential dropout rate of approximately 30%, we aimed to recruit at least 180 participants. Ultimately, 187 were enrolled (Table 2).

**Table 2.** Summary of our study and comparable digital cognitive intervention studies.

	Our study	Research 1 (Ahn and Park, 2016 [59])	Research 2 (Campbell et al, 2020 [60])	Research 3 (Chapman et al, 2021 [61])	Research 4 (Nakada et al, 2024 [62])	Research 5 (Laane et al, 2023 [63])
Study design	Single-arm	Single-arm	Single-arm	Single-arm	Single-arm	Single-arm
Sample size	187	100	103	145	132 (pilot)	180
Effect size	Hedges *g*= 0.5	N/A^a^ (moderate improvement)	N/A (focus on correlations)	N/A (improvement reported)	N/A (improvement reported)	N/A (improvement reported)
*P* value	<.05	<.05	<.05	<.05	<.05	<.05

a N/A: not applicable.

The demographic profile showed that out of 133 participants, 69 (51.9%) were female, 119 (89.5%) had been married, and 51 (38.3%) held elementary school diplomas. Among them, out of 133 participants, 91 (68.4%) reported living with a spouse (Table 3). No missing data were observed for the baseline demographic variables, including cognitive function score (K-MMSE-2), depression score (SGDS-K), social support measure (MOS-SSS), or participation measure (TWEETS), among the participants who were included in the final analysis. Participants who had incomplete outcome data (n=54) were excluded from the analysis.

**Table 3.** Demographic characteristics of the participants (N=133).

Demographic characteristics	Values
Age (years)	
Mean (SD)	64.76 (6.76)
50‐59, n (%)	32 (24.1)
60‐69, n (%)	67 (50.4)
70‐79, n (%)	34 (24.6)
Sex, n (%)	
Male	64 (48.1)
Female	69 (51.9)
Marital status, n (%)	
Married	119 (89.5)
Unmarried	14 (10.5)
Education, n (%)	
No education	2 (1.5)
Elementary school diploma	51 (38.3)
Middle school diploma	33 (24.8)
High school diploma	30 (22.6)
Community college diploma	3 (2.3)
College diploma and professional degree	14 (13.4)
Cohabitation type, n (%)	
Living alone	5 (3.8)
Married couple	91 (68.4)
Married couple with children	31 (23.3)
Living with relatives other than spouse/children (eg, siblings) or with nonrelatives	6 (4.5)

### Primary Result

#### Overall Change in Cognitive Function

**Figure 4.**
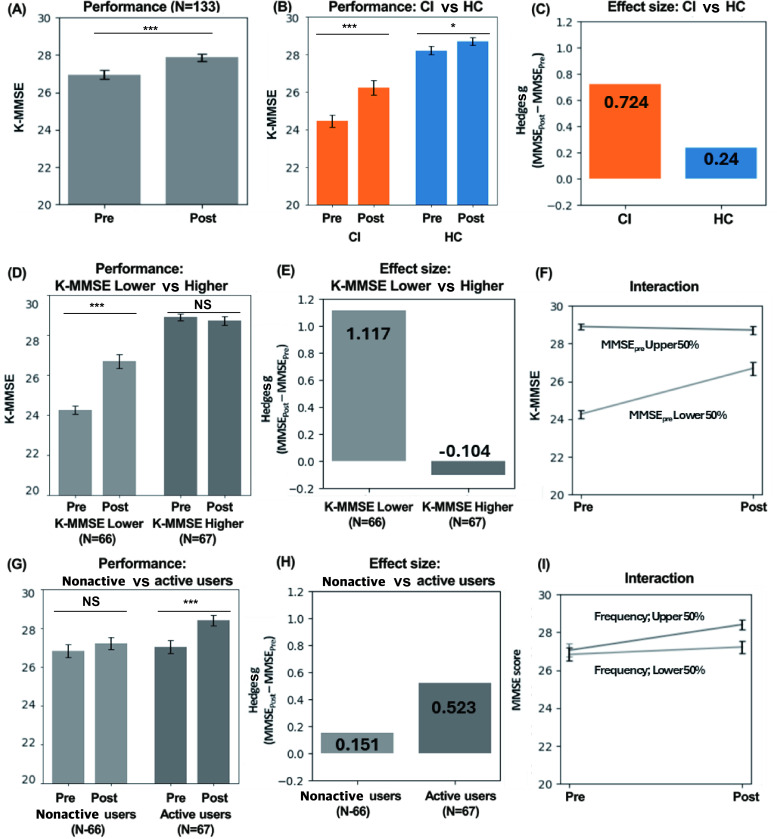
Overall results. (A) Pre- and postintervention comparison of cognitive function. (B) Performance enhancement comparing participants in the cognitive impairment (CI) risk group (K-MMSE-2 scores 19‐23) versus those in the HC group (K-MMSE-2 scores ≥24). (C) Effect size. (D) Performance comparison between participants below and above baseline K-MMSE-2 median scores. (E) Effect size. (F) Interaction effect. (G) Performance comparison between low (nonactive) and high (active) app usage frequency groups (based on end-of-experiment user logs). (H) Effect size. (I) Interaction effect. We note that “K-MMSE” in the plot refers to K-MMSE-2 (independent samples *t* test; *P<.05,**P<.01,***P<.001; note: K-MMSE-2 baseline values were adjusted from 0 to 23‐26 for readability; error bars indicate standard error of the mean). Orange bars indicate the CI group, blue bars indicate the HC group, and gray bars represent overall or subgroup comparisons. CI: cognitive impairment; HC: healthy control; K-MMSE-2: Korean-Mini Mental State Examination—Version 2; MMSE: Mini Mental State Examination; NS: not significant.

For all 133 participants, K-MMSE-2 scores showed significant improvement after using the *Care & Cure* program (Figure 4A; pretrial: mean 26.95, SD 2.77; posttrial: mean 27.87, SD 2.46; *t*_132_=−4.106, *P*<.001) with a weak effect size (Hedges *g*=0.350). The cognitive impairment risk group included 15 participants with baseline K-MMSE-2 scores ranging from 19 to 23, corresponding to the K-MMSE-2 normative range for mild cognitive impairment (18-23). The healthy control (HC) group included 118 participants with baseline scores of 24 or higher, corresponding to the cognitively normal range in the K-MMSE-2 norms. The cognitive impairment group showed greater improvement in cognitive function than the HC group (Figure 4B; cognitive impairment: *P*<.001; HC: *P*<.05). Effect sizes for each group are presented in Figure 4C. The cognitive impairment group demonstrated a strong effect size (Hedges *g*=0.724), whereas the HC group showed a weak effect (Hedges *g*=0.240).

#### Performance Changes in K-MMSE-2 Subject to Baseline Cognitive Function

Similar patterns emerged between groups categorized by baseline K-MMSE-2 median scores. For sensitivity analysis, we divided participants into 2 groups based on their baseline K-MMSE-2 scores: the lower K-MMSE-2 group (n=66) and the higher K-MMSE-2 group (n=67). Participants whose K-MMSE-2 scores fell below the baseline median formed a lower group, while the remaining participants were assigned to the higher group.

Independent samples *t* tests examined cognitive improvement differences between groups. The analysis showed that the K-MMSE-2 lower group demonstrated significant score increases posttreatment (independent *t* test; *P*<.001), whereas the K-MMSE-2 higher group showed no statistically significant changes (independent *t* test; *P*<.241) (Figure 4D). Effect size analysis showed substantial impact in the K-MMSE-2 lower group (Hedges *g*=1.117), while the higher group displayed an ignorable negative effect (Hedges *g*=−0.104; Figure 4E). The interaction effect between baseline cognitive level and time is presented in Figure 4F.

#### Performance Changes in K-MMSE-2 Based on the Application Usage Frequency

The active user group (n=67) showed significant increases in K-MMSE-2 scores after the intervention, whereas the nonactive user group (n=66) did not show statistically significant changes in K-MMSE-2 scores (independent *t* test; *P*<.001) (Figure 4G). The lower frequency group (nonactive group) showed a weak effect (Hedges *g*=0.151), while the higher frequency group demonstrated a moderate effect (Hedges *g*=0.523; Figure 4H). The interaction effect between usage frequency and time is presented in Figure 4I.

#### Performance Differences in K-MMSE-2 According to Age

To investigate the influence of age, we again divided participants once more into 2 age groups based on the median age (66 years): the lower age group (age ≤66 years; n=70) and the higher age group (age>66 years; n=63). Independent *t* tests examined cognitive improvement differences using K-MMSE-2 Delta scores. Both age groups showed increased K-MMSE-2 Delta scores, although these changes lacked statistical significance (lower age group: mean 0.96, SD 2.27 years; higher age group: mean 0.87, SD 2.90 years; *t*_131_=−0.187, *P* value not applicable). Effect size analysis revealed negligible differences between groups (Hedges *g*=0.03). See Figure S2 in Multimedia Appendix 1 for more details.

### Secondary Result

#### Impact of Group Intervention on Engagement, Social Support, and Cognitive Function

To investigate the impact of group chat usage and cognitive training on cognitive function, simple regression analyses were conducted on 2 separate occasions. In the initial regression model, usage of the *Our Town* (group chat), measured as the number of interactions, significantly influenced usage of cognitive training (*F*_1,131_=153.646, *P*<.001). The explanatory power of the regression model was approximately 54.0% (*R*²=0.540). The Durbin-Watson statistic was 1.502, which is close to 2, indicating no significant concerns regarding the assumption of independence of residuals. Regarding the significance of the regression coefficients, engagement in *Our Town* (group chat) demonstrated a statistically significant positive effect on the usage of cognitive training (*β*=.735, *P*<.001). This finding suggests a positive and significant relationship between increased engagement in the group chat and greater usage of cognitive training tools.

**Figure 5.**
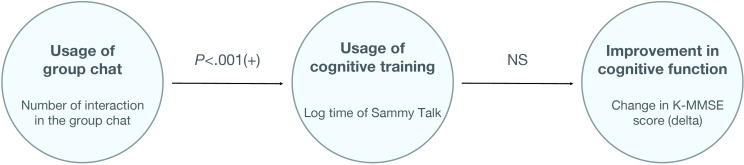
Impact of group intervention on engagement and cognitive function. K-MMSE-2: Korean-Mini Mental State Examination—Version 2; NS: not significant.

In the second regression model, usage of the cognitive training tools, measured as total log time, did not significantly influence improvement in cognitive function, as assessed by the K-MMSE-2 Delta scores (*F*_1,131_=0.158, *P*=.692). The explanatory power of the second regression model was minimal, accounting for only 0.1% (*R*²=0.001). The Durbin-Watson statistic was 2.050, which is close to 2, indicating no significant concerns regarding the assumption of independence of residuals. Regarding the significance of the regression coefficients, cognitive training usage did not statistically significantly influence cognitive function improvement (*β*=−.035, *P*=.692). This finding suggests that cognitive training usage did not contribute to cognitive function enhancement (Figure 5 and Table 4).

**Table 4.** Summary of regression models examining group chat usage, cognitive training, and cognitive function.

	Model	*B*	SE	*β*	*t* test (*df*)	*P* value	*F* test (*df*); *P* value	*R* ^2^	Durbin-Watson (95% CI)
1	Group chat usage → cognitive training usage	1.138	0.092	.735	12.395 (131)	<.001	153.646 (1,131); <.001	0.54	1.138(0.956 to 1.320)
2	Cognitive training usage → cognitive function improvement	−0.001	0.003	−.035	−0.397 (131)	.69	0.158 (1,131); .69	0.001	-0.001(-0.007 to 0.005)

We conducted additional regression analyses to examine how social support mediated the relationships between group chat usage, cognitive training, and cognitive function improvement. In the initial regression model, *Our Town* usage (measured by the number of interactions) significantly influenced cognitive training usage (*F*_1,131_=153.646, *P*<.001). The explanatory power of the regression model was approximately 54.0% (*R*²=0.540). The Durbin-Watson statistic was 1.502, which is close to 2, indicating no significant concerns regarding the assumption of independence of residuals. Regarding the significance of the regression coefficients, engagement in *Our Town* (group chat) demonstrated a statistically significant positive effect on the usage of cognitive training (*β*=.735, *P*<.001). This finding suggests a clear positive relationship between increased engagement in the group chat and greater usage of cognitive training tools.

In the second regression model, frequent cognitive training usage, defined as total interaction time with the conversational agent, significantly influenced social support improvement, as indicated by social support scores (*F*_1,131_=16.830, *P*<.001). The explanatory power of the second regression model was 11.4% (*R*²=0.114). The Durbin-Watson statistic was 1.946, which is close to 2, indicating no significant concerns regarding the assumption of independence of residuals. Regarding the significance of the regression coefficients, frequent engagement with the conversational agent demonstrated a statistically significant positive effect on social support improvement (*β*=.337, *P*<.001). This finding suggests that participants who interacted more frequently with the conversational agent reported greater perceived social support.

In the third regression model, improvements in social support had a statistically significant effect on cognitive function enhancement, as measured by the K-MMSE-2 Delta scores (*F*_1,131_=6.830, *P*<.05). The explanatory power of the regression model was 4.4% (*R*²=0.044). The Durbin-Watson statistic was 2.084, which is close to 2, indicating no significant concerns regarding the assumption of independence of residuals. Regarding the significance of the regression coefficients, higher social support scores were associated with greater improvements in cognitive function (*β*=.211, *P*<.05). This finding suggests that participants with strong social support experienced greater gains in cognitive function.

**Figure 6.**
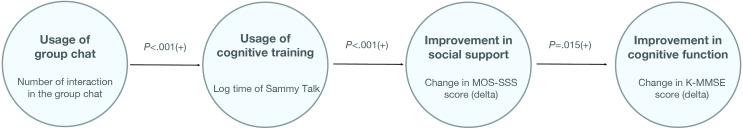
Impact of group intervention on engagement, social support, and cognitive function (as a part of path analysis). K-MMSE: Korean-Mini Mental State Examination; MMSE: Mini Mental State Examination.

Conversely, cognitive training usage did not have a statistically significant impact on cognitive function enhancement (*P*=.692). These findings suggest that cognitive function does not improve when individuals use cognitive games as a stand-alone intervention. However, combining cognitive games with group interventions that increase social support and motivation leads to cognitive function improvement (Figure 6 and Table 5).

**Table 5.** Summary of regression analyses examining the mediating role of social support.

	Model	*B*	SE	*β*	*t* test (*df*)	*P* value	*F* test (*df*); *P* value	*R* ^2^	Durbin-Watson (95% CI)
1	Group chat usage → cognitive training usage	1.138	0.092	.735	12.395 (131)	<.001	153.646 (1,131); <.001	0.540	1.138(0.956 to 1.320)
2	Cognitive training usage → social support improvement	0.061	0.015	.337	4.102 (131)	<.001	16.830 (1,131); <.001	0.114	0.061 (0.031 to 0.091)
3	Social support improvement → cognitive function improvement	0.034	0.014	.211	2.467 (131)	<.05	6.084 (1,131); <.05	0.044	0.034(0.006 to 0.062)

#### Change in Social Support, Willingness to Participation, and Depression

Social support was divided into four categories: (1) emotional or informational, (2) material, (3) affectional, and (4) positive social interaction support. Emotional or informational support showed the highest pre-post difference with statistical significance (*t*_132_=−6.509, *P*<.001) and a moderately strong effect size (Hedges *g*=0.70). Willingness to participate was assessed across 3 dimensions: affect, behavior, and cognition. Affect captured participants’ emotional attitudes toward their involvement, such as expressing enjoyment when using *Sammy Talk* for cognitive training or showing enthusiasm about interacting with other members in *Our Town*. Next, behavior referred to observable participation such as attendance or task engagement, including frequency of playing arithmetic and memory games in *Sammy Talk* or actively posting messages and responding to others in *Our Town* social platform. Finally, cognition reflected participants’ beliefs and understanding of the program’s value, such as recognizing the *Sammy Talk*’s cognitive exercises could improve their mental abilities or believing that social interactions in *Our Town* could enhance their well-being and reduce isolation. Willingness to participate demonstrated significant differences in cognitive factors (*t*_132_=2.159, *P*<.05) with a nearly moderate effect size (Hedges *g*=0.43). Within the subcategories of willingness to participate, affect showed significant pre-post differences (*t*_132_=2.008, *P*<.05) with a moderate effect size (Hedges *g*=0.51), whereas behavioral measures showed no significant differences (*t*_132_=0.244, *P*=.81). Depression levels exhibited significant pre-post differences (*t*_132_=3.093, *P*<.01) with a weak effect size (Hedges *g*=0.21). Table 6 presents these results in summary.

**Table 6.** Comparison of social support, emotional relief with program use before and after.

Parameters	Week 0 (n=133), mean (SD)	Week 12 (n=133), mean (SD)	Hedges *g*	*t* test (*df*)	*P* value
Social support					
Emotional/informational support	30.19 (7.04)	34.10 (5.43)	0.70	−6.509 (132)	<.001
Material support	15.18 (3.78)	16.25 (3.06)	0.37	−3.345 (132)	<.01
Affectionate support	10.81 (2.86)	11.87 (2.56)	0.29	−4.256 (132)	<.001
Positive social interaction support	15.23 (3.76)	16.43 (3.00)	0.37	−3.803 (132)	<.001
Willingness to participation					
Behavior	10.45 (3.60)	10.35 (3.77)	0.47	0.244 (132)	.81
Cognition	12.08 (3.34)	11.27 (3.58)	0.43	2.159 (132)	<.05
Affect	10.78 (3.90)	9.90 (4.02)	0.51	2.008 (132)	<.05
Depression					
SGDS-K^a^	2.77 (2.59)	2.23 (2.13)	0.21	3.093 (132)	<.01

a SGDS-K: Short Form Geriatric Depression Scale—Korean version.

#### Engagement Analysis: Sammy Talk and Our Town

**Figure 7.**
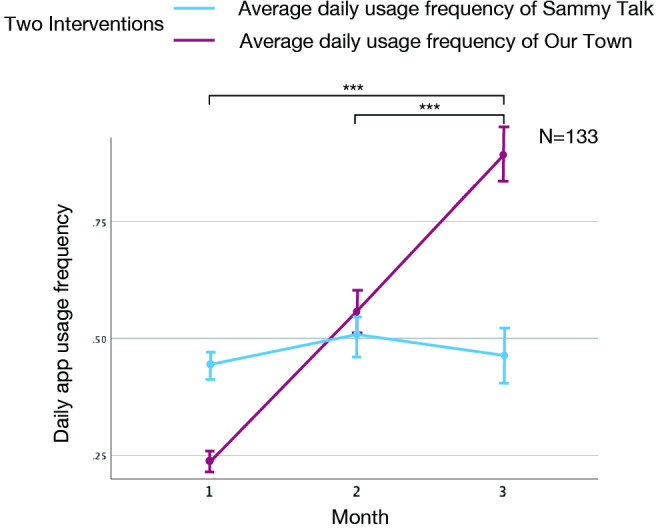
Changes in participant *Sammy Talk* and *Our Town* usage frequency following intervention implementation. *P<.05,**P<.01,***P<.001.

To examine the engagement patterns, we conducted a repeated-measures ANOVA on the average daily usage frequency of *Sammy Talk* (conversation-based intervention) and *Our Town* (community-based intervention) over 3 months (Figure 7). The analysis showed significant differences in average daily usage frequency between *Sammy Talk* and *Our Town* during the third month (*P*<.001). Results indicated a significant main effect of usage duration (*F*_2,264_=20.282, *P*<.001) and a significant interaction between intervention types (eg, *Sammy Talk* and *Our Town*) and usage duration (*F*_2,264_=17.859, *P*<.001). *Our Town* demonstrated steadily increasing average daily usage, which reached the highest in month 3 (mean 0.90, SD 0.11), followed by month 2 (mean 0.56, SD0.08) and month 1 (mean 0.24, SD 0.05). In contrast, *Sammy Talk* usage frequency varied, with highest engagement observed in month 2 (mean 0.52, SD 0.08), followed by month 3 (mean 0.47, SD0.11) and month 1 (mean 0.42, SD 0.05).

## Discussion

### Principal Findings

The *Care & Cure* program demonstrated effectiveness in improving cognitive function across all 133 participants, with particularly strong enhancement among individuals with lower K-MMSE-2 scores. This aligns with the program’s evidence-based development, which incorporated clinical expertise from neurologists at Mokdong Ewha Womans University Hospital.

The *Sammy Talk* content followed validated clinical frameworks across multiple cognitive domains. The calculation games were derived from the *WRAT-3 Arithmetic subset* and *Digit Symbol Substitution Test* [41,42]. Visuospatial tasks incorporated the *Mental Rotation Test* [43], whereas attention-focused games used crossword puzzles and the *Montreal Cognitive Assessment letter—a tapping test* [44,45]. Executive function games were adapted from the *University of California, San Diego Performance-Based Skills Assessment* (*UPSA-3*) and the *Trail Making Test* [46,47]. Memory tasks integrated the *Korean Naming Test*, *Chinese idioms*, and *Rey-Osterrieth Complex Figure Test* [48-51]. Language games incorporated the *Montreal Cognitive Assessment* initial letter fluency test and *Scrambled Sentences Test* [44,52]. However, the specific cognitive impact of individual game components remains to be clarified in future research.

Participants with lower baseline K-MMSE-2 scores (hereafter referred to as the lower group) showed larger effect sizes, as they had greater potential for measurable improvement. By contrast, those with higher baseline scores (hereafter referred to as the higher group) showed little change, having already approached their cognitive performance ceiling. This difference reflects a well-known ceiling effect among cognitively normal participants. Previous studies examining short-term interventions have consistently reported that people with cognitively normal conditions achieve only limited gains, whereas those with cognitive impairment show greater improvement [64-66]. Moreover, participants in the lower group faced a higher risk of decline and therefore benefited more from the clinically validated content designed by neurologists to target multiple cognitive domains, including calculation, attention, language, memory, visuospatial ability, and executive function. This finding is consistent with previous research using validated clinical tasks, which confirmed that participants in the lower group achieved greater improvements than those in the higher group [64,65].

Higher usage frequency of *Care & Cure* corresponded with enhanced cognitive function. *Our Town* appeared to moderate this effect by increasing *Sammy Talk* engagement. Participants who engaged more often in *Our Town* engagement dedicated more time to *Sammy Talk* and achieved superior cognitive function scores. The group chat format likely enhanced engagement through shared performance goals, achievement reports, and peer encouragement. These findings align with previous research on group chat–based cognitive enhancement programs [31,32].

Nonetheless, *Our Town* did not directly enhance cognitive function; rather, its impact emerged through social support mechanisms. The integration of *Our Town* and *Sammy Talk* (a software robot) in the *Care & Cure* program enhanced social support, which in turn improved cognitive function. While *Our Town* did not bring about direct cognitive benefits, it served as an indirect motivational means by sending consistent reminder messages to participants. Participants exchanged greetings and shared daily experiences in *Our Town* while learning practical knowledge and conversation-related vocabulary through *Sammy Talk*. This encouraged more frequent communication both online and offline. These repeated interactions in turn created a social support network through daily greetings, experience sharing, and conversational practice. The AI chatbot provided additional support by offering affective encouragement and fostering positive exchanges, leading participants to report higher social support scores. This finding aligns well with previous studies demonstrating that AI chatbots deliver social support and reduce depressive symptoms among older adults [67,68]. Social support and emotional stability function as preventive factors that preserve cognitive function by reducing loneliness and chronic stress, both recognized contributors to memory decline and executive dysfunction [67-71]. Conversations and social interactions demand memory recall, language use, and problem-solving, repeatedly stimulating cognitive processes and strengthening cognitive reserve [72-74].

On a secondary issue, the *Care & Cure* program effectively reduced emotional distress, with participants experiencing emotional changes through enhanced social support. Program engagement, measured through *Our Town* chat interactions, showed increasing participant responses to the chatbot-based software agent, reaching 3.8 times the initial level by month 3 (Figure 6). This increased engagement suggested more frequent conversations with both the software agent and other participants, potentially facilitating positive social exchanges and providing emotional, affectionate, and informational support. However, chat interaction frequency alone provides limited insight into intervention adherence. Future research should examine more precise adherence measurements. The study suggests that increased social support influenced participants’ depressive symptoms [75-78].

### Postanalysis on Dropout

Nearly 3 out of 10 participants (28.8%) dropped out of the study, with education emerging as the primary differentiating factor. Education, rather than age or sex, accounted for this difference, as people who completed the intervention had significantly more years of education than those who dropped out (*t*_131_=2.43, *P*=.016). These results show that education level, which reflects accumulated social and cognitive resources, was associated with dropout, whereas age and sex were not. Participants with limited education and digital literacy often struggled to use the digital cognitive training program. Lower educational attainment, which often limits digital literacy, reduced participants’ ability to use messenger applications and type messages, preventing them from joining group chats or completing training tasks [18,20,21]. Those who lacked primary school education and could not perform basic arithmetic operations were unable to complete calculations tasks and eventually dropped out. Participants with very low education levels may therefore be more likely to drop out of digital cognitive training programs [18,20,21]. Although not statistically significant, males were likely to drop out more frequently than females, suggesting that future studies should consider strategies to improve male retention (see Multimedia Appendix 1, Section VI for more details).

### Limitations

Despite the significant improvement in participants’ cognitive capacity through the use of our proposal in general, the research has some limitations to address. First, the single-arm design without a control group reduces confidence in attributing changes to the intervention. Without a comparison group, it would be very difficult to establish that observed improvements resulted solely from the intervention, as changes might be able to reflect natural progression, practice effects from repeated testing, or placebo effects. In addition, it would be challenging to determine whether improvements are clinically meaningful compared with standard care or alternative interventions. Second, participant interaction with the software robot in *Our Town* (the group chat component) was lower than anticipated, with its primary effect manifesting through increased usage of *Sammy Talk*. Third, the sample was drawn from a single midsize Korean city with relatively homogeneous demographic characteristics, limiting the generalizability of findings to other regions or populations. Fourth, the program’s reliance on *KakaoTalk* as the delivery platform might restrict applicability beyond the Korean context. These methodological considerations highlight areas for future investigation, including randomized controlled trials with appropriate control groups, multisite studies across diverse populations, and head-to-head comparisons with standard assessment tools to establish clinical superiority.

### Conclusions and Future Research Direction

The *Care & Cure* program demonstrated effectiveness for cognitive enhancement, with more frequent participation corresponding to greater improvement. The program showed particular efficacy among cognitively impaired participants. Additional benefits included reduced depressive symptoms, enhanced social support, and increased participation willingness.

Our hypothesis proposed that increased *Our Town* usage would enhance *Sammy Talk* engagement, subsequently improving cognitive function. The data confirmed that higher *Our Town* usage directly increased *Sammy Talk* participation. However, increased *Sammy Talk* usage alone did not yield statistically significant cognitive improvements. When examining social support as a mediator between *Sammy Talk* usage and cognitive enhancement, results indicated that social support directly influenced cognitive improvement. This relationship requires further investigation to understand underlying mechanisms.

While the top 50% of program participants showed greater K-MMSE-2 improvements, the causal relationship between *Sammy Talk* usage and cognitive enhancement lacked statistical significance. Future research should examine the relationship between *Our Town* and *Sammy Talk* usage patterns while addressing the methodological issues raised in this study. Controlled trials with comparison groups are necessary to establish causal effects. For example, a potential future study could compare an intervention group using both *Sammy Talk* and *Our Town*, with a control group attending a traditional face-to-face dementia prevention program. Researchers should also test strategies to increase participation in group chat functions, which serve as sources of social support [79-81]. Gamification approaches, such as small financial rewards, goal setting, or competition elements, may enhance motivation to engage [82,83]. Additionally, context-based reminders drawing on Ecological Momentary Interventions or Ecological Momentary Assessments could encourage more consistent use of both *Sammy Talk* and *Our Town* [84-86].

Next, future studies had better recruit participants from varied regions and sociodemographic groups to extend findings beyond a single Korean midsized city. For instance, researchers could target English-speaking populations from middle-class and low-class households or urban groups with higher digital literacy and educational attainment [87,88]. Moreover, researchers should conduct longitudinal follow-up studies that track participants for at least 1 year to examine whether cognitive benefits persist and to determine whether digital training combined with social support prevents long-term cognitive decline [89,90]. For example, participants could use the intervention for 1 year or more, with outcomes measured at regular intervals to evaluate intervention effectiveness over time. Finally, since this program relied on *KakaoTalk* as its delivery platform, which limits generalizability beyond Korea, future studies should better adapt the same model to other messaging services that provide chatbot Application Programming Interfaces, such as *WeChat*, *WhatsApp*, or *Facebook Messenger*, to test how the intervention performs across different cultural and technological contexts [91,92].

Finally, future works should also take into account education as a covariate, which requires stratified randomization and sensitivity analyses to ensure the robustness of findings. Researchers should also design tasks that assess cognition without relying on arithmetic, so that individuals without formal schooling can participate fully in the longitudinal intervention. Finally, incorporating competitive gamification elements, which may appeal particularly to males who often demonstrate stronger achievement motivation than females, could enhance digital program retention by using leaderboards or quizzes that provide modest rewards and recognize performance [93-95]. By pursuing these directions, future research would be able to clarify how digital cognitive training and social interaction interplay with each other, and identify practical strategies that strengthen engagement and broaden applicability across populations.

## Supplementary material

10.2196/75308 Multimedia Appendix 1 Cognitive functions software robot study.

## Abbreviations

AI: artificial intelligence
HC: healthy control
IRB: institutional review board
K-MMSE-2: Korean-Mini Mental State Examination—Version 2
MOS-SSS: Medical Outcomes Study–Social Support Survey
SGDS-K: Short Form Geriatric Depression Scale—Korean Version
TWEETS: Twente Engagement with eHealth Technologies Scale
UPSA-3: University of California, San Diego Performance-Based Skills Assessment
