# Supplementary material for: Enhancing Cognitive Functions of Older Adults With Software Robot: Longitudinal Exploratory Field Study
Source: JMIR Mhealth Uhealth. 2026 Jun 24;14:e75308. doi: 10.2196/75308 (PMC13293570; doi:10.2196/75308)
Supplement: Multimedia Appendix 1 [file mhealth-v14-e75308-s001.docx]

## **Multimedia Appendix 1**

## **I. The City of Suncheon**

## The city of *Suncheon*, a mid-sized city in *South Jeolla Province*, South Korea with a population of approximately 280,000. The city's elderly population reflects South Korea's aging demographics, with about 20% of residents aged 65 years or older [1].

## *Suncheon* is well known for its robust infrastructure to support senior citizens through welfare centers, nursing homes, and silver towns that provide housing and healthcare services for older adults [2].

## The city combines urban and rural characteristics, with a robust agricultural and fishing sector coexisting alongside expanding urban development. This dual character creates a balanced environment, offering residents the benefits of both rural and modern urban living [3,4].

## **II. Suncheon Nonghyup**

## *Suncheon Nonghyup*, a key institution in the city, provides substantial support to the elderly community. The *National Agricultural Cooperative Federation (NACF)*, known locally as *Nonghyup*, operates over 1,100 branches nationwide and delivers essential services to farmers and rural communities [5]. As a prominent local cooperative, *Suncheon Nonghyup* serves approximately 30,000 registered members, offering a wide range of services, including financial support, agricultural resources, and welfare programs that particularly benefit the region's older adults [6].

## We recruited participants through this institution in partnership with Haii Inc., the engineering and development company that developed the *Care & Cure* program, under a Memorandum of Understanding (MOU). This partnership aimed to establish a welfare system integrating digital healthcare services for older adults in the region. We chose *Suncheon Nonghyup* as our recruitment partner due to its extensive welfare network and its role as the largest cooperative in the area, making it ideal for efficiently recruiting participants for this digital healthcare program.

## **III. The Rationale for Age Group Selection**

## We focused on adults aged 55 to 75, as this age range represents a critical transition period characterized by early cognitive changes varying levels of digital technology adoption. Mild cognitive impairment (MCI) often emerges during this period [7], making it a crucial phase for implementing and evaluating digital cognitive enhancement strategies [8].

## While digital literacy levels vary within this population, many individuals in this age group are increasingly adopting digital technologies [9]. This growing adoption presents an opportunity to deliver scalable interventions. By targeting this specific age range, we evaluated the effectiveness of digital training during a period when adults are both cognitively vulnerable and adapting to digital tools.

**IV. Description on 15 Cognitive Training Games in *Sammy Talk***

The 15 cognitive training games in *Sammy Talk* target multiple cognitive domains to enhance overall cognitive function in older adults. The program includes:

**Calculation games**: Improve mathematical reasoning and problem-solving abilities through repetitive arithmetic operations.

**Attention games**: Promote selective attention and visual concentration by requiring users to identify specific words and shapes.

**Memory games**: Stimulate both short-term memory (information processing and temporary retention) and long-term memory (extended information retention) through activities such as poem recitation, flag matching, and object location tasks.

**Language games**: Enhance language processing and vocabulary through sentence completion and error correction activities.

**Executive function games**: Strengthen planning and execution abilities through rule-based card sorting and item selection tasks.

**Visuospatial games**: Improve spatial awareness and visual information processing by requiring users to identify differences between images.

The games automatically adjust their difficulty levels based on the user’s performance, providing consistent cognitive stimulation and promoting continuous learning.

**Table S1**. Sammy Talk contents and cognitive domain.

| Cognitive Domain | Game Name | Game Description | Name of evidence-based clinical examination |
| --- | --- | --- | --- |
| Calculation | Step by Step From the Basics | A game where numbers and operators are combined to perform calculations. | WRAT-3 Arithmetic Subset |
|  | Guess the Number | A game where players match the number of characters to the given equation. | WRAT-3 Arithmetic Subset |
|  | Try to Break the Code | A game where players decipher an expression using a password and to guess the answer. | Digit Symbol Substitution Test (DSST) |
|  | What Should We Eat? | A game where a player selects a menu item, calculates the total cost, and determines the change. | WRAT-3 Arithmetic Subset |
| Visuospatial | What’s the Difference? | A game where a player compares four images and finds the one that is different. | Mental Rotation Test (MRT) |
| Attention | Let’s Find a Word! | A game where a player identify the correct word for a topic from a dial mixed with multiple letters | Crossword Puzzle |
|  | Let’s Find the Same Picture! | A game where a player counts the number of matching pictures described in a given sentence. | 1) MoCA - Letter a Tapping Test  2) Impossible Spotter |
| Executive Function | Let’s Make a Travel Plan | A game where a player schedules a trip without exceeding a given budget and time | A Three Subtest Short Form of the UCSD Performance-Based Skills Assessment (UPSA) |
|  | Guess the Right Order | A game where a player arranges tasks in the correct order. | Trail Making Test |
| Memory | Let’s Memorize the Poem | A game where a player memorizes a poem and then does a memory test on the poem. | Remembering a List |
|  | Match a Flag with the Country! | A game where a player links a flag photo to its corresponding country name. | Korean Naming Test (KNT) |
|  | Study an Idiom | After studying four-character idioms, a player solves two types of related questions | Chinese Idioms |
|  | Remember the Position of the Fruit | A task where a player recalls the position of visual stimuli within a grid. | Rey-Osterrieth Complex Figure Test |
| Language | Guess the First Letter | A game where a player sees a word missing its first letter and identifies the common first letter. | MoCA -  Initial Letter Fluency |
|  | Let’s Reorganize the Letters | A game where a player rearranges presented letters to form a complete word. | Scrambled Sentences Test (SST) |

**V. Scales**

**The Korean-Mini Mental State Examination-Version 2 (K-MMSE)**

The study used the K-MMSE-2 to measure cognitive function. This test assesses the degree of dementia and comprises the following domains: time, place, memory registration, attention and calculation, memory association, language, and visuospatial capital. The total score for the six domains is 30 points. A score of 24 or more indicates cognitive normality, 18-23 indicates mild cognitive impairment, and 17 or less indicates severe cognitive impairment. K-MMSE-2 was developed by Kown [10], as a tool suitable for use with Korean older adults, and it is most widely used in clinical settings. Previous studies have reported an interrater reliability of 0.96 and test-retest agreement of 0.86 for this measurement [11].

**Short form Geriatric Depression Scale-Korean Version (SGDS-K)**

Short form Geriatric Depression Scale-Korean Version (SGDS-K) has demonstrated high reliability and agreement compared with other scales. Yesavage created this scale to address the unique characteristics of depressive disorders in older adults, which differ from those in younger age groups [12]. The SGDS-K is a brief and simple test for face-to-face assessment of many participants in a short amount of time [13]. This study builds on previous research that highlights how cognitive enhancement programs significantly impact depression among community-dwelling older adults [14].

**The Medical Outcomes Study-Social Support Survey (MOS-SSS)**

This study used the Korean version of MOS-SSS, a social support test [15]. This test includes items grouped into four factors: material support, emotional support, positive interaction support, and emotional/informational support [16]. Participants rate the items on a 5-point scale ranging from 'never' to’ always,’ with total scores ranging from 19 to 95. Higher scores indicate greater social support. Social support is a psychosocial factor, influencing health outcomes [17,18]. This study aimed to measure social support to enhance the quality of life among the older adults by implementing interventions designed to improve cognitive function and promote positive emotional effects.

**The Twente Engagement with eHealth Technologies Scale (TWEETS)**

TWEETS is a test developed to measure engagement in eHealth programs. It includes nine items that assess three constructs: behavior, cognition, and affect. TWEETS was developed based on interviews with users, addressing the limitations of existing methods for measuring engagement in eHealth programs. Most studies measure user engagement by analyzing the frequency of log data from the system. However, this method inadequately captures engagement for only a small portion of users without considering their subjective experience [19]. To address this issue, this study aims to measure user engagement not only through users' program log data, but also through a questionnaire that captures users' subjective willingness to engage. This approach compensates for the limitations of log data and accounts for users' subjective experiences.

**Participants’ Log Data on the Use of Applications**

This study collected the users’ game performance rate from the *Sammy* administrative website to measure engagement through log data. Additionally, it recorded the number of speech bubbles uttered within *Our Town* for each individual and group to measure the degree of interactivity among group members, as in previous studies. In group chats on messaging applications such as *KakaoTalk* or *WhatsApp*, "speech bubbles" refers to the visual representation of text-based exchanges among participants, similar to their function on other social media platforms. The total number of speech bubbles was used as a metric to quantify the frequency of interactions within the group chat. This provided a quantitative measure to message volume, offering a valuable indicator of user engagement and social interaction during the intervention.

**VI. Post-hoc Analysis on Drop-out**

**Sex.** Competitors included total n=133 (64 men and 69 women), whereas non-completers included total n=54 (30 men and 24 women). The distribution, which we tested with a chi-square analysis, did not differ significantly by completion status (χ²(1)=0.85, p=0.360). Within the non-completer group, 55.6% (30/54) were men and 44.4% (24/54) were women.

**Age.** Non-completers, who had a mean age of 67.50 years (SD=5.93), were slightly older than completers, whose mean age was 66.00 years (SD=6.76). This difference, which we tested with Welch’s t-test, was not statistically reliable (t=1.50, *P*=0.135).

**Education.** Non-completers, whose average years of education corresponded to middle-school level (M=8.30, SD=2.68), had fewer years of schooling than completers, whose average corresponded to high-school level (M=9.47, SD=3.62). This difference, which we again tested with Welch’s t-test, reached statistical significance (t=2.43, *P*=0.016).

**Table S2**. Comparison of completers and non-completers.

| Variable | Non-completers (n=54) | Competitors (n=133) | Test statistic | *P* value |
| --- | --- | --- | --- | --- |
| Sex, n (%) | Male: 30 (55.6%) Female: 24 (44.4%) | Male: 64 (48.1%) Female: 69 (51.9%) | χ²(1)=0.85 | *P* = 0.360 |
| Age (years),  mean (SD) | 67.50 (5.93) | 66.00 (6.76) | t=1.50 | *P* = 0.14 |
| Education level, mean (SD) | 8.30 (2.68) | 9.47 (3.62) | t=2.43 | *pP*= 0.016 |

**a** Education coding: 4=elementary dropout, 6=elementary graduate, 9=middle school graduate, 10=high school dropout, 12=high school graduate, 16=college graduate, 18=postgraduate.

**VII. References**

1. Suncheon City Government. Suncheon demographics and city profile. Suncheon: Suncheon City Government; 2023. Available from: <https://www.suncheon.go.kr>
2. Korea Statistical Information Service (KOSIS). Aging population in South Jeolla Province. Daejeon: Korea National Statistical Office; 2022. Available from: <https://kosis.kr>
3. Ministry of Health and Welfare. Infrastructure for elderly welfare in South Korea. Seoul: Ministry of Health and Welfare; 2023. Available from: <http://www.mohw.go.kr>
4. Jeonnam Provincial Office. Rural and urban development in Suncheon. Jeonnam Provincial Office; 2022. Available from: <https://www.jeonnam.go.kr>
5. National Agricultural Cooperative Federation (NACF). Nonghyup annual report 2023: Branches and services. Seoul: NACF; 2023. Available from: <https://www.nonghyup.com>
6. Ministry of Agriculture, Food and Rural Affairs. Rural welfare and cooperative services in South Korea. Seoul: Republic of Korea Government Publication; 2022. Available from: <http://www.mafra.go.kr>
7. Petersen RC, Lopez O, Armstrong MJ, et al. Practice guideline update summary: Mild cognitive impairment. Neurology. 2018 Jan 16;90(3):126-135. [doi: [10.1212/WNL.0000000000004826](https://www.neurology.org/doi/10.1212/WNL.0000000000004826)] [Medline: [29282327](https://pubmed.ncbi.nlm.nih.gov/29282327/)]
8. Pappas MA, Demertzi E, Papagerasimou Y, Koukianakis L, Voukelatos N, Drigas A. Cognitive-based e-learning design for older adults. Social Sciences 2019;8(1):6. [doi:[10.3390/socsci8010006](https://www.mdpi.com/2076-0760/8/1/6)]
9. Anderson M, Perrin A. Tech adoption climbs among older adults. Pew Research Center. 2017 May 17. Available from: <https://www.pewresearch.org/internet/2017/05/17/tech-adoption-climbs-among-older-adults/>
10. Kwon, Yong-Cheol, & Park, Jong-Han. A standardization study of the Korean version of the Mini-mental state examination for the elderly (MMSE-K). Neuropsychiatry. 1989: 28(1), 125-135.
11. Folstein, M F et al. Mini-mental state". A practical method for grading the cognitive state of patients for the clinician. Journal of psychiatric research vol. 12,3 1975: 189-98. [doi: [10.1016/0022-3956(75)90026-6](https://linkinghub.elsevier.com/retrieve/pii/0022395675900266)] [Medline: [1202204](https://pubmed.ncbi.nlm.nih.gov/1202204/)]
12. Yesavage, Jerome A., et al. Development and validation of a geriatric depression screening scale: a preliminary report. Journal of psychiatric research 17.1 1982: 37-49. [doi: [10.1016/0022-3956(82)90033-4](https://linkinghub.elsevier.com/retrieve/pii/0022395682900334)] [Medline: [7183759](https://pubmed.ncbi.nlm.nih.gov/7183759/)]
13. Cho, M. J., Bae, J. N., Suh, G. H., Hahm, B. J., Kim, J. K., Lee, D. W., & Kang, M. H. Validation of geriatric depression scale, Korean version (GDS) in the assessment of DSM-III-R major depression. Journal of Korean Neuropsychiatric Association. 1999: 38(1), 48-63.
14. Won Kyung-ah, Lee Jong-hoon and Kim Jung-ran. Effects of Cognitive Health Lifestyle Improvement Program for Cognitive Function, Depression, and Health behavior in Community-dwelling Elderly. The Journal of Korean Society of Cognitive Rehabilitation 8.2 2019: 59-74. [[FREE Full text](https://alz-journals.onlinelibrary.wiley.com/doi/epdf/10.1016/j.jalz.2019.09.011)] [doi: [10.1016/j.jalz.2019.09.011](https://alz-journals.onlinelibrary.wiley.com/doi/10.1016/j.jalz.2019.09.011)]
15. Lim, Min-Kyoung, et al. Social support and self-rated health status in a low income neighborhood of Seoul, Korea. Korean journal of preventive medicine 2003: 54-62. [[FREE Full text](https://www.jpmph.org/upload/pdf/jpmph-36-1-54.pdf)]
16. Alonso Fachado, Alfonso, et al. Adaptação cultural e validação da versão portuguesa: Questionário medical outcomes study social support survey (MOS-SSS). 2007. [[FREE Full text](about:blank)] [Medline: [18331696](https://pubmed.ncbi.nlm.nih.gov/18331696/)]
17. Cohen S. 1988. Psychosocial models of the role of social support in the etiology of physical disease. Health Psychology 7(3): 269. [doi: [10.1037/0278-6133.7.3.269](https://psycnet.apa.org/doiLanding?doi=10.1037%2F0278-6133.7.3.269)] [Medline: [3289916](https://pubmed.ncbi.nlm.nih.gov/3289916/)]
18. Berkman LF, Leo-Summers L, Horwitz RI. 1992. Emotional support and survival after myocardial infarction prospective, population-based study of the elderly. Annals of Internal Medicine 117(12): 1003-1009. [doi: [10.7326/0003-4819-117-12-1003](https://www.acpjournals.org/doi/10.7326/0003-4819-117-12-1003)] [Medline: [1443968](https://pubmed.ncbi.nlm.nih.gov/1443968/)]
19. Yardley, Lucy, et al. Understanding and promoting effective engagement with digital behavior change interventions. American journal of preventive medicine 51.5 2016: 833-842. [doi: [10.1016/j.amepre.2016.06.015](https://linkinghub.elsevier.com/retrieve/pii/S0749379716302434)] [Medline: [27745683](https://pubmed.ncbi.nlm.nih.gov/27745683/)]


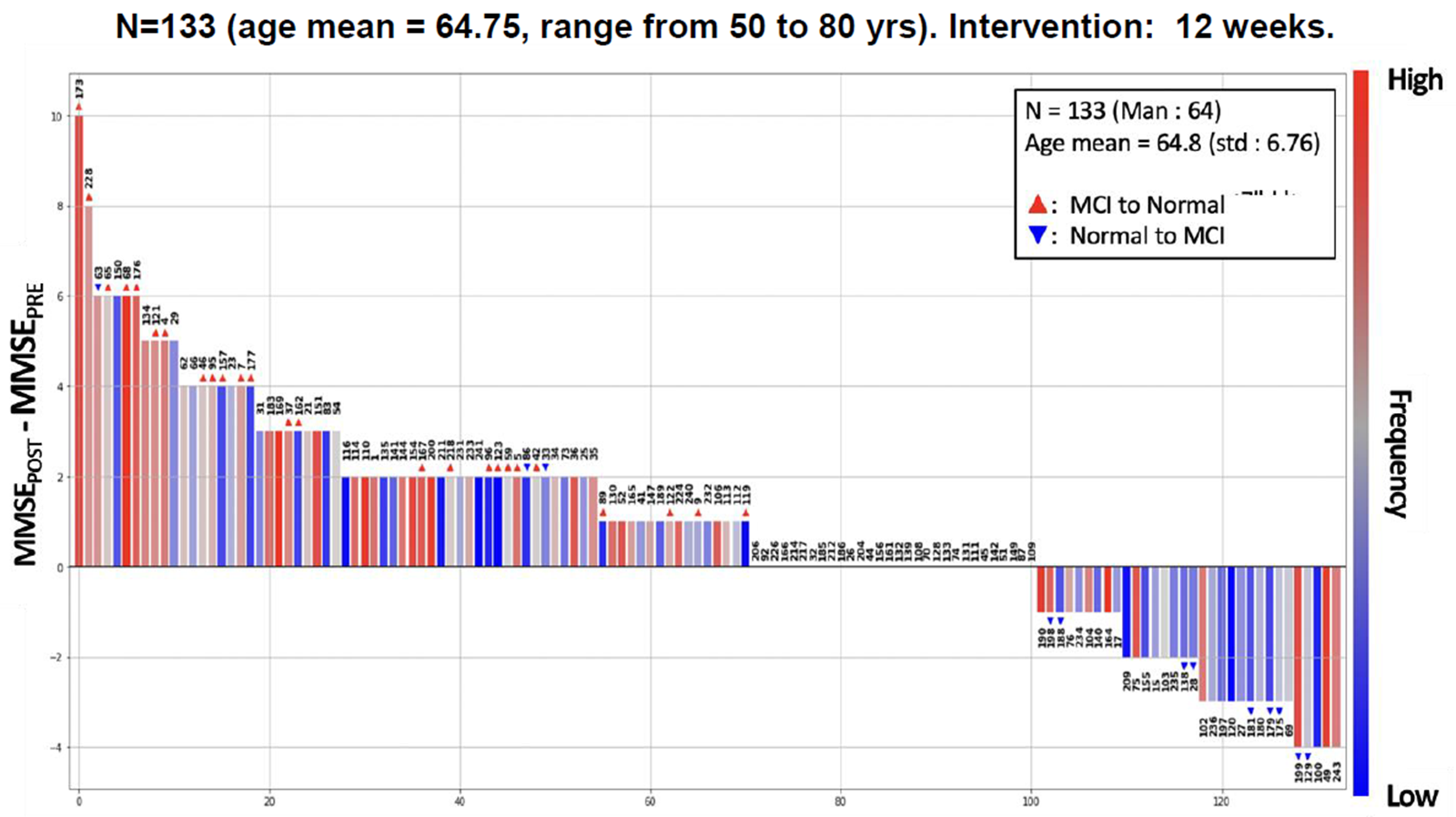


**Figure S1.** Cognitive function changes among participants based on usage frequency.

We examined whether demographic characteristics, particularly age and marital status, could have confounding effects. As suggested, we conducted subgroup analyses by dividing participants into lower and higher groups based on the median age of 67 years. Participants aged ≤ 67 were categorized as the lower age group, and those aged > 67 as the higher age group. As shown in Supplementary Figure 2, no significant differences were observed between the two groups. In addition, we compared baseline K-MMSE-2 scores according to marital status, which showed no significant difference between unmarried participants (M=27.29, SD=2.92, n=14) and married participants (M=26.92, SD=2.76, n=119), t=0.45, *P*=0.658. Therefore, we found no evidence that age and marital status acted as confounding factors in our regression analysis.


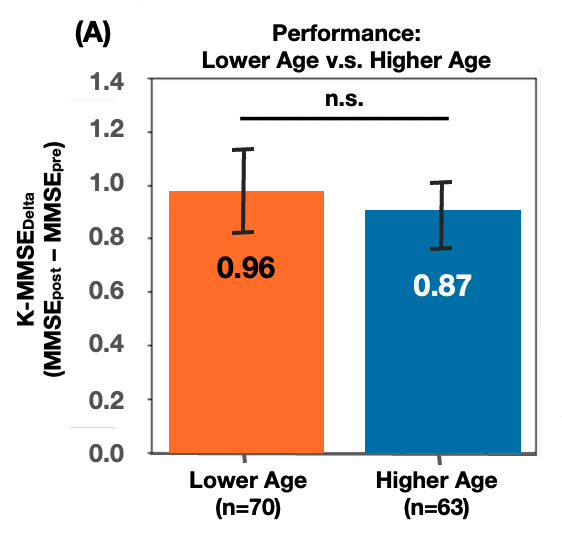


**Figure S2.** K-MMSE-2 Delta comparisons between lower and higher age groups (**p* < .05, ***p* < .01, ****p* < .001; The error bars represent the Standard Error of the Mean (SEM)).


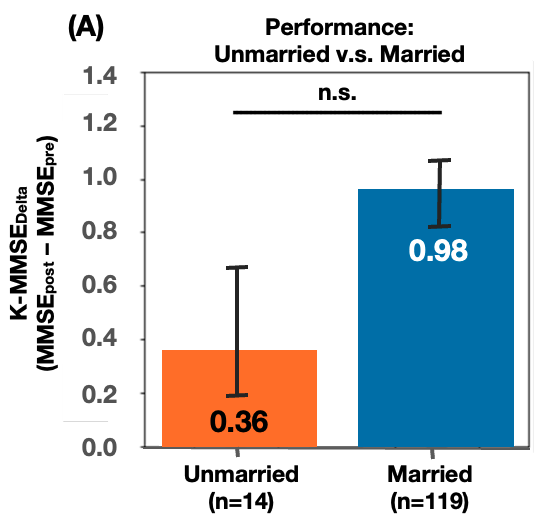


**Figure S3.** K-MMSE-2 Delta comparisons between unmarried and married groups (**p* < .05, ***p* < .01, ****p* < .001; The error bars represent the Standard Error of the Mean (SEM)).

Participants in this study received guidance to engage with *Sammy Talk* games three times a day. The analysis showed lower engagement than recommended, with participants averaging 0.47 game sessions and 2.34 minutes of daily use. The group chat service, *Our Town*, showed similar patterns of limited engagement. Participants averaged 0.56 daily interactions and approximately one minute of daily usage in group chat interactions with the agent or other members. These patterns indicate lower frequency and duration of engagement than the study guidelines suggested.

**Table S3.** Usage adherence for Sammy Talk and Our Town

| Category | Ave. Daily Usage Frequency of *Sammy* | Ave. Daily Usage Time of *Sammy* | Ave. Daily Usage Frequency of *Our Town* | Ave. Daily Usage Time of *Our Town* |
| --- | --- | --- | --- | --- |
|  | n = 133 | n = 133 | n = 133 | n = 133 |
| Mean | 0.47 game session  / Day | 2 minute 20 second / Day | 0.56 Time  / Day | 50 second  / Day |
| SD | 0.95 | 4.76 | 1.02 | 1.53 |
